# Antifreeze Protein Prolongs the Life-Time of Insulinoma Cells during Hypothermic Preservation

**DOI:** 10.1371/journal.pone.0073643

**Published:** 2013-09-17

**Authors:** Tatsuro Kamijima, Mami Sakashita, Ai Miura, Yoshiyuki Nishimiya, Sakae Tsuda

**Affiliations:** 1 Bioproduction Research Institute, National Institute of Advanced Industrial Science and Technology (AIST), Sapporo, Hokkaido, Japan; 2 Graduate School of Life Science, Hokkaido University, Sapporo, Hokkaido, Japan; Iwate University, Japan

## Abstract

It is sometimes desirable to preserve mammalian cells by hypothermia rather than freezing during short term transplantation. Here we found an ability of hypothermic (+4°C) preservation of fish antifreeze protein (AFP) against rat insulinoma cells denoted as RIN-5F. The preservation ability was compared between type I–III AFPs and antifreeze glycoprotein (AFGP), which could be recently mass-prepared by a developed technique utilizing the muscle homogenates, but not the blood serum, of cold-adapted fishes. For AFGP, whose molecular weight is distributed in the range from 2.6 to 34 kDa, only the proteins less than 10 kDa were examined. The viability rate was evaluated by counting of the preserved RIN-5F cells unstained with trypan blue. Significantly, either AFPI or AFPIII dissolved into Euro-Collins (EC) solution at a concentration of 10 mg/ml could preserve approximately 60% of the cells for 5 days at +4°C. The 5-day preserved RIN-5F cells retained the ability to secrete insulin. Only 2% of the cells were, however, preserved for 5 days without AFP. Confocal photomicroscopy experiments further showed the significant binding ability of AFP to the cell surface. These results suggest that fish AFP enables 5-day quality storage of the insulinoma cells collected from a donor without freezing.

## Introduction

Cells obtained by either cultivation or extraction from human or animal tissues are used in the fields of regenerative medicine and livestock farming [1]. For short-term transplantations occurring within 1–5 days, it is desirable to preserve the cells without freezing. We generally handled such cells as assemblies of several to millions, and the percentage of viable cells in the total number (i.e., survival rate) is generally improved by soaking them in a preservation solution comprising inorganic salts, glycerol, sugars, etc., such as lactated Ringer [2], Euro-Collins (EC) [3], and the University of Wisconsin (UW) solutions [4]. The performance of each solution is highly dependent on the cell-type, and varies widely from cell to cell, even as a function of their age [1]. Indeed, the above solutions were initially developed to preserve specific organs, but are now used for virtually every type of cell and organ. The EC- and UW-solutions could, for example, preserve human hepatocytes under hypothermic conditions (e.g. +4°C) for 24 to 72 h [5], [6]. Here we examined whether the performance of a cell-preservation solution is improved by addition of fish antifreeze protein (AFP), for which a general cell-membrane protection ability has been recognized in the last two decades [7].

AFPs, first extracted from blood sera of polar fishes in the 1970s, were initially identified as macromolecules that specifically adsorb onto ice crystals to inhibit their growth [8]. The AFPs were categorized into AFPI–IV and antifreeze glycoprotein (AFGP), according to their differences in amino acid sequence and tertiary structure [9], [10]. AFPI is an amphipathic α-helical peptide (M.w. = 3.5 kDa). AFPII is an elongated globular protein with mixed secondary structures stabilized with disulfide bonds (M.w. = 14 kDa). AFPIII is made up of short β-strands and one helical turn, which constructs a flat-faced globular fold (M.w. = 6.5 kDa). AFPIV consists of four α-helices of similar length which are folded in to a four-helix bundle. AFGP is made up of repeating tripeptide units (Ala-Thr-Ala)*_n_* to form a polyproline type II helix, whose Thr is modified with a disaccharide moiety. A polar fish, such as Antarctic Nototheniids, expresses eight AFGPs ranging in size from 2.6 to 34 kDa, where AFGP7 and 8 are less than 10.5 kDa. For AFPs and AFGP, their ice-binding function can be identified by their ability to shape ice crystals (ex. Into hexagonal bipyramids). The ability of ice-binding is also characterized with thermal hysteresis (TH), which is a difference between the non-colligative freezing point depression and the elevated melting point [11].

The cell-preservation ability of fish AFPs was first reported by Rubinsky and colleagues in 1990 [12]. They revealed that AFGP protected the structural integrity of the oolemma of pig oocytes, and inhibited ion leakage across the oolemma for 24 h at +4°C. The best preservation result was obtained with a solution of phosphate buffered saline (PBS) containing AFGP at a concentration of 40 mg/ml. Rubinsky et al. further showed that the fish AFPI–III can also protect the membrane of immature bovine oocytes for 24 h at 4°C, when AFPs were dissolved at a concentration of 20 mg/ml [13]. The ability to protect whole rat liver against hypothermic damage was also identified for AFPIII [14]. Although such cell-protection abilities of fish AFPs were significant, their marginal performance with a specific cell-line, and their individual differences in cell-preservation ability were not clarified. One of the reasons for this was the scarcity of fish AFPs, which had to be purified from blood sera of polar fishes.

Recently, a simple method of purifying massive amounts of fish AFP1–III and AFGP was developed [15]. This uses muscle homogenates of mid-latitude fishes as the source material. The present study used fish-muscle-derived AFPs supplied by a food company. The AFPs dissolved into EC-solution were examined for their ability to prolong the life-time of rat insulinoma cell-line RIN-5F under hypothermic conditions.

## Materials and Methods

Rat insulinoma cell line RIN-5F (CRL-2058) was purchased from ATCC (http://www.atcc.org/). For hypothermic preservations at 4°C, we prepared solutions of medium-A, medium-B, and trypsin-A according to [16]. Their components are as follows: medium A, RPMI-1640 medium (ATCC, 30-2001) containing 10% of fetal bovine serum (FBS) (ATCC, 30-2020); medium B: the RPMI-1640 medium containing 1% of FBS; trypsin A: 0.25% bovine trypsin plus 1 mM of EDTA dissolved in phosphate buffered saline (PBS, 0.2 g/l of KCl, 0.2 g/l of KH_2_PO_4_, 8 g/l of NaCl, and 2.9 g/l of Na_2_HPO_4_-12H_2_O).

**Figure 1:**
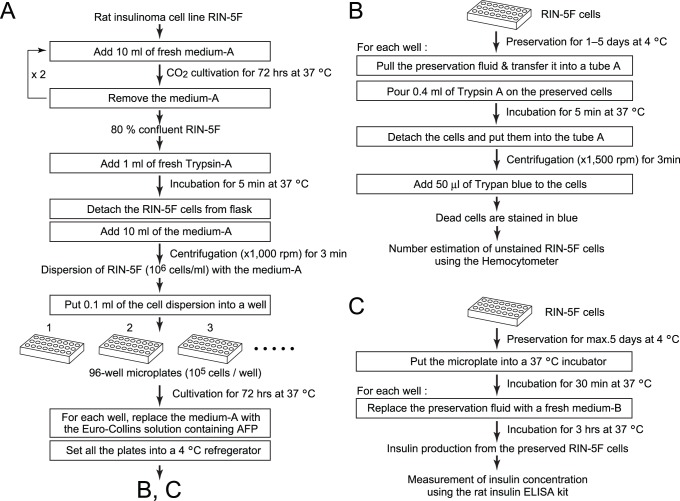
Flow-charts showing the preparation of RIN-5F cells and their survival rate evaluation. A. Preparation of RIN-5F cells before starting 1–5-day preservation experiments at +4°C. B. Procedures to estimate the number of living cells using the hemocytometer. C. Procedures to measure the amount of insulin secreted from the living cells. Medium-A consists of RPMI-1640 medium containing 10% of fetal bovine serum (FBS). Medium-B consists of the medium containing 1% of FBS. Trypsin-A consists of 0.25% bovine trypsin plus 1 mM of EDTA dissolved in phosphate buffered saline (PBS).

The preservation experiment on RIN-5F cells was divided into three steps: (a) setting of the cells, (b) 1–5-day preservation with a solution containing AFP, and (c) survival rate evaluation. Figure 1A shows a flow-chart of step (a). We first cultured the cells in a CO_2_ incubator with medium-A in a flask by performing 2 cycles of 72 hr-cultivation at 37°C to reach an 80%-confluent state. The cells were successively detached from the flask by the addition of 1 ml of trypsin-A, and put into a centrifuge tube with 10 ml of medium-A. Following centrifugation at 1,000×g for 3 min, the collected cells were suspended in medium-A to a concentration of approximately 10^6^ cells/ml. An aliquot (100 µl) of this cell-suspension was put into each well of a 96-well microplate such that each well had approximately 10^5^ of RIN-5F cells. Following incubation for 3 days at 37°C, medium-A was carefully removed from each well so as not to disturb the cells. Subsequently, 100 µl of the AFP-containing solution, which was pre-cooled at 4°C, was put into each well to start the 1–5-day preservation experiments. We prepared 3–5 microplates for each set of experiments.

We chose EC-solution as a base fluid to dissolve the AFP as it contains no protein, which simplified the basis for the protein-induced effect on the cells. The purified samples of AFPI from *Liopsetta pinnifasciata*, AFPII from *Hypomesus japonicus*, AFPIII from *Zoarces elongates Kner*, and AFGP from *Eleginus gracilis* were provided from Nichirei Foods Inc. (9-Shinminato, Mihama-ku, Chiba-shi, Chiba 261–8545, Japan), and were used without further purification. These fishes were captured from mid-latitude sea areas near Japan, and their minced muscles were utilized for the source material to purify mass-amount of each AFP [15]. The AFP sample was dissolved into a freshly made EC-solution consisting of 99.3 mM of KCl, 15.1 mM of KH_2_PO_4_, 9.0 mM of NaHCO_3_, and 194 mM of glucose (pH = 7.4), whose osmolarity was 355 mM/kg of H_2_O [5]. Bovine serum albumin (BSA) and trehalose were also separately dissolved into the EC solution to evaluate their cell-preservation abilities.

The survival rate of RIN-5F cells was examined by protocols shown in Figure 1B. After 24, 72, and 120 h of preservations at 4°C, we carefully withdrew the EC-solution containing AFP from a well, and stored it in a 1.5 ml tube. An aliquot (0.04 ml) of trypsin-A was then poured onto the cells remaining in the well. Following a 37°C-incubation for 5 min, the cells were detached and added back into the AFP-solution withdrawn from that well into the tube, before being centrifuged at 1,500×g for 3 min. The collected cells were re-suspended with 0.04% of trypan blue dissolved in PBS. The number of unstained living cells was counted using a hemocytometer, and a ratio of the viable cells versus the total number of the cells determined before preservation, was defined as the survival rate (%). Such evaluations were performed for 3 microplates taken out after 24, 72, and 120 h of preservation.

The ability of the RIN-5F cells to produce insulin was also evaluated (Figure 1C) before and after the preservation. For the latter evaluation, we took out a 96-well microplate from the 4°C-incubator after 120 h of preservation, and settled it into a 37°C-incubator for 30 min. The AFP-solution was then replaced with fresh medium-B, and the plate was further incubated at 37°C for 3 h to induce the secretion of insulin. We then evaluated a ratio of the secreted amount of insulin after the 120 hrs of preservation versus the amount measured before the preservation, which was examined as another survival rate. The concentration of the secreted insulin was estimated using a rat insulin ELISA kit (Shibayagi Co. Ltd.).

The antifreeze activities of AFPs were examined as described in [17] by using an in-house photomicroscope system with a Leica DMLB 100 photomicroscope (Leica Microsystems AG, Wetzlar, Germany) equipped with a Linkam LK600 temperature controller (Linkam, Surrey, UK). We monitored the AFP-induced change of the ice crystal shape (eg. hexagonal bipyramid), and also the ice growth initiation- and melting- temperatures to evaluate thermal hysteresis, a measure of antifreeze activity.

A Leica DM IRE2 confocal microscope system was also used to observe a human hepatoma cell, HepG2, and RIN-5F cells in the EC-solution containing AFPIII or BSA. The two proteins were labeled with a fluorescent detergent, Alexa-488 (Invitrogen, USA), which binds to lysine. All of the cells were incubated with 0.4 mg/ml of each protein solution for 1 hr at 37°C. The photomicroscope image was then captured after rinsing with the solution without protein. All the images and movies were recorded using a color-video 3CCD camera (Sony, Tokyo, Japan).

## Results

**Figure 2:**
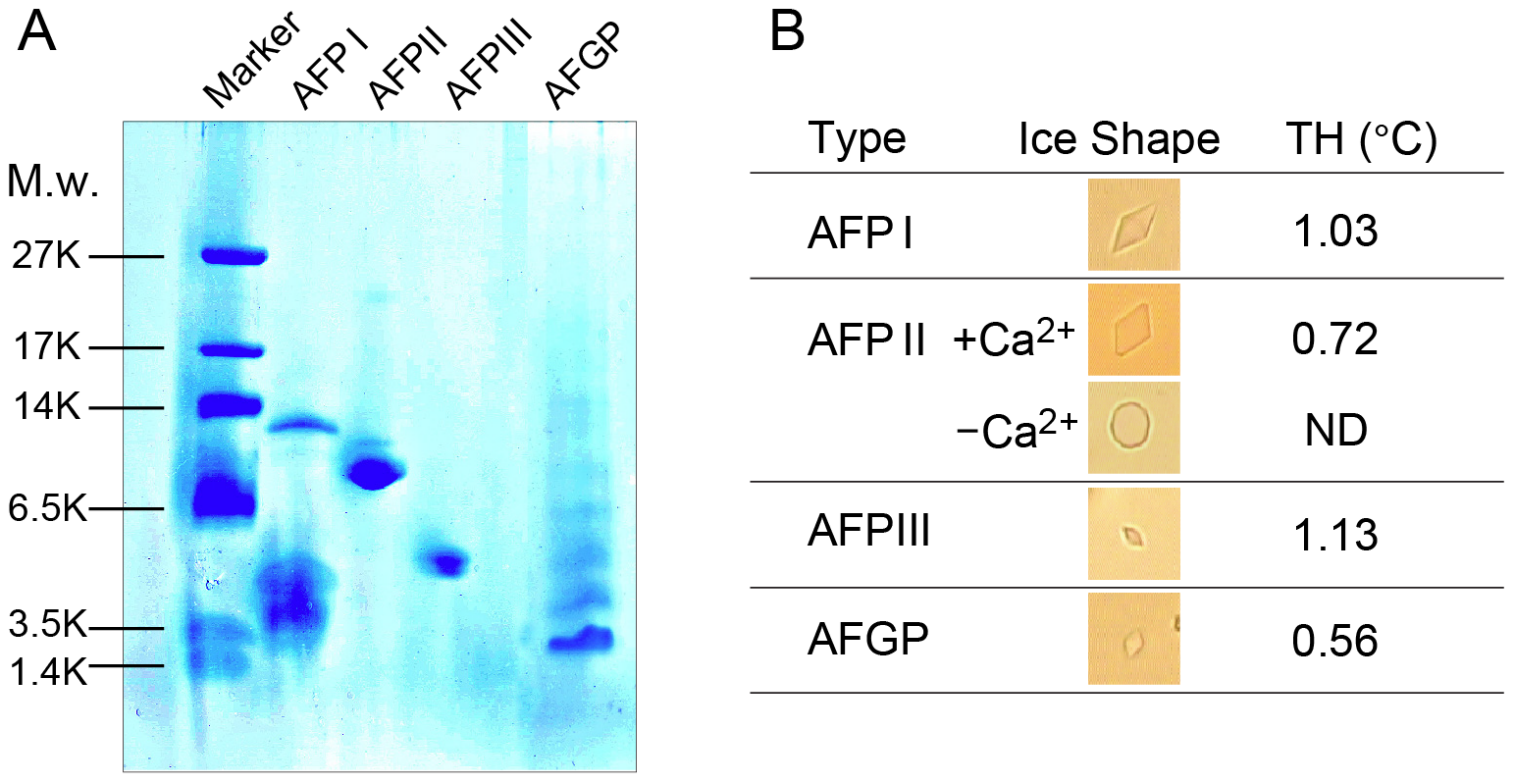
Analyses of purity and structural integrity of the AFP samples. A. Electrophoretogram of AFPI–III and AFGP samples provided by Nichirei Foods Inc. on 16.5% tricine-SDS-PAGE. The AFGP sample only contains molecules less than 10 kDa owing to a filtration step. B. The shape of an ice crystal and TH activity obtained for each AFP sample dissolved into the Euro-Collins solution. All protein concentrations were 10 mg/ml.

Purity of the AFPI –III and AFGP samples provided from Nichirei Foods Inc. was confirmed by SDS-PAGE (Figure 2). Each AFP is a natural mixture of the isoforms. For example, the 6.5 kDa AFPIII from *Zoarces elongates Kner* consists of at least 13 isoforms whose ice-binding activities are different [18]. As shown in Figure 2A, the purified AFPIII migrated on the gel to a position between bovine trypsin inhibitor (6 kDa) and insulin (3 kDa) as reported previously [19]. The AFGP sample comprises the polypeptides less than 10 kDa, whose main species were assignable to AFGP7 and 8 [20].

The structural integrity of each AFP in the cell-preservation solution was confirmed by observations of their ice-shaping ability and TH activity. The samples of AFPI, AFPII plus Ca^2+^-ion, AFPIII, and AFGP can each shape an ice crystal into the hexagonal bipyramid (Figure 2B), indicating that they form native structures in the EC-solution to exert their functions. AFPII requires a Ca^2+^ ion for ice-binding activity [21], so that the “unshaped” disk-like ice crystal was observed in the absence of Ca^2+^ (Figure 2B). Detection of approximately 1°C of TH activity for AFPI–III also indicated the functionality of their native structures. The lower TH activity of the AFGP sample (0.56°C) can be ascribed to the lack of higher M.w species, AFGP1–5 (>10 kDa).

The survival rate of RIN-5F cells was examined for seven EC-solutions, five of which contains 10 mg/ml of the proteins, AFPI, AFPII, AFPIII, AFGP, and BSA. The 10 mg/ml concentration was chosen, since it was the optimal concentration for another mammalian cell [5], and also the solubility limit of the present AFPIII sample. For AFPII, unbound Ca^2+^ was removed since excess Ca^2+^ ion is harmful to the cells. The survival rate was also examined with plain EC solution as the control. A well-known cell-protection agent, trehalose, was also examined. It was dissolved into the EC solution without glucose to 70 mg/ml to adjust its osmorality to that of the other solutions. Approximately 10^5^ of RIN-5F cells prepared in AFPI–III, AFGP, trehalose, and BSA solutions were put into four wells of a 96-well microplate (Figure 1). At least four sets of these microplates were prepared for each time-point of preservation (0, 24, 72, and 120 h), and we repeated each set of the experiments three times. The number of the living cells were hence averaged over 3×4 wells for one preservation period, and the number at 0 h was used as a denominator to evaluate the survival rate (%) with a standard deviation.

**Figure 3:**
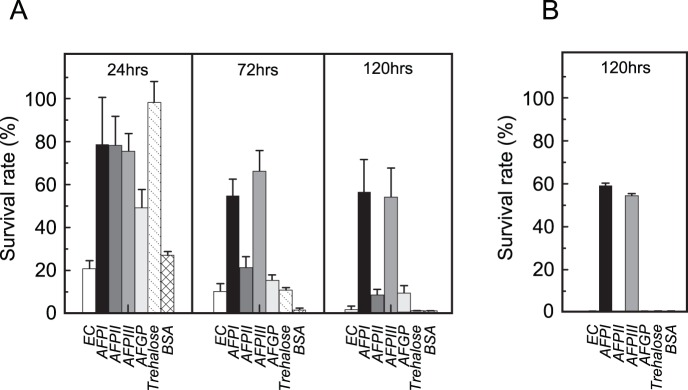
Time-dependent change in the survival rate (%) of rat insulinoma cell-line, RIN-5F, under hypothermic exposure (+4°C). A. The survival rate evaluated by counting the number of living cells (Fig. 1B). B. The survival rate evaluated by measuring the amount of insulin secreted from living cells (Fig. 1C). A freshly made Euro-Collins solution (EC) was used as a basic cell-preservation fluid to dissolve AFPI–III, trehalose, and BSA.

After 24 h of exposure to 4°C (Figure 3A), trehalose could keep alive 98% of RIN-5F cells, while EC solution preserved only 20% of the cells. BSA gave a slightly better result (38%) than EC. The survival rate with the three AFPs was similar at 78%. The rate with AFGP was lower (49%) compared with the other AFPs.

After 72 h of preservation, approximately 90% of the cell-population died in both EC and the trehalose solution. The survival rates with AFPII and AFGP also decrease to 22 and 18%, respectively. However, there was almost 0% survival of the cells in the BSA solution. In contrast, much greater survival rates of 56% and 68% were obtained for AFPI and III, respectively.

After 120 h of preservation, a 58% survival rate was obtained with AFPI, implying that there is practically no change in the rate for AFPI from 72 h to 120 h. Similarly AFPIII keeps its preservation ability, although it went down slightly to 57%. The protective ability of AFPI and III was further verified by measuring the concentration of insulin secreted from RIN-5F cells before and after 120 h of preservation (Figure 3B). As shown, approximately 60% of the cells retain the ability to secrete insulin, but only when the EC solution contained either AFPI or III.

**Figure 4:**
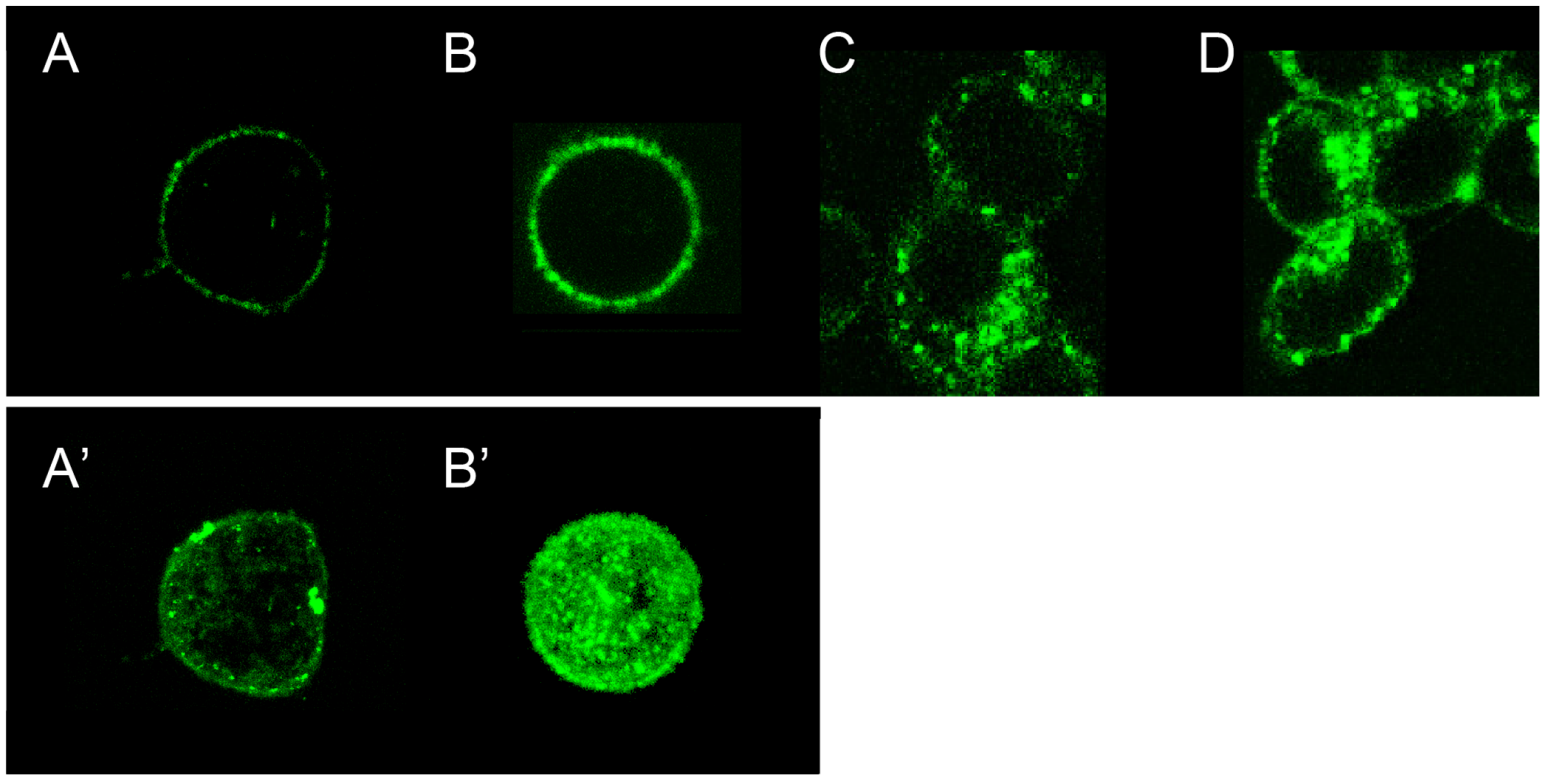
Confocal photomicroscope images of human hepatoma cells (HepG2) and rat insulinoma cells (RIN-5F) in Euro Collins-solutions containing 0.4 mg/ml of BSA (A), AFPIII (B and C), and AFPI (D) labeled with fluorescence. The images A and B show the slice data of 1 µm-width of HepG2, and A’ and B’ an intact cell images synthesized by stacking of the slices. These 4 images were reproduced from [22] with permission. The images C and D are the slice data for RIN-5F cells. All the images were captured after 1 h-preservation at 37°C. Accumulation of AFPIII on the cell-surface is more evident compared with BSA.

Figure 4 shows confocal photomicroscope images of HepG2 and RIN-5F cells examining the membrane-protection ability of AFP. Images A and B show the slice images of 1 micrometer width of a HepG2 cell, and images C and D are those of RIN-5F cells, respectively. Images A–D were captured after 1 h preservation at 37°C in EC-solution containing BSA (A), AFPIII (B and C), and AFPI (D). All of the proteins were labeled with the fluorescence reagent, Alexa-488, which binds to lysine. Note that BSA is a 583-residue protein and contains 20 lysines. In contrast, only one lysine exists in the 65-residue AFP III and in the 37-residue AFPI [8]. As can be seen, surface potion of the cell incubated with AFPIII (Fig. 4B) is brightened significantly compared from that with BSA (Fig. 4A). These data suggests that AFPIII molecules bind uniformly onto the cell surface, while BSA undergoes heterogeneous binding. The superior binding ability of AFPIII is more evident by comparison of the intact cell images (Fig. 4A’ and 4B’), each of which were synthesized by stacking of the slices. For RIN-5F cells (Fig. 4C and 4D), it was difficult to capture the noncongestion image, as they tend to stick together and collapsed very easily.

## Discussion

The observed life time elongation of RIN-5F cells could be attributed to the content of AFP in the EC solution since the solution itself provided very little protection to the cells. AFP I and III at concentrations of 10 mg/ml could keep alive approximately 60% of a insulinoma cell-line for 120 h, and the preserved cells retained the ability to secrete insulin. Previous studies showed that AFPI–III could preserve oocytes for 24 h at 4°C; e.g. 50% of bovine immature oocytes were able to undergo *in vitro* maturation after preservation [13]. This is consistent with the present results; approximately 78% survival rate was obtained after 24 h of preservation with AFPI–III. The observed protective ability of AFGP was, however, much poorer compared with the others, which became consistently evident during the preservation period (Figure 3). This is probably due to the present AFGP sample containing only the smaller glycopeptides of less than 10 kDa (Figure 2). The full set of AFGP peptides were also needed for the preservation of pig oocytes [12]. Significantly, the preservation ability of AFPII became poorer than that of AFPI and III after 72 h at the low temperature. Such a difference in ability was not visible in 24–48 h preservation period, but became evident at a time of marginal performance, 120 h, in this case.

The mechanism by which AFPs protect cells under hypothermia is not well understood. In general, the lipid bilayer is in a fluid state at physiological temperatures. As the temperature is lowered, the bilayer segregates and becomes leaky, which allows ions from the exterior to enter into the cell in uncontrollable ways, leading to the cell destructions [1]. Rubinsky showed the ability of AFPI and AFGP to block the passive ion channels [23], which was assumed to reduce the leakiness of membranes and provide cold-tolerance to the cells. Tomczak et al. showed that AFP is introduced into the lipid bilayer through a hydrophobic interaction [24]. This interaction increases the phase transition temperature of the membranes and alters the molecular packing of the acryl chains, leading to a reduction in membrane permeability. A recent time-lapse SECM experiment further demonstrated that a human hepatoma cell, HepG2, was swollen and ruptured during hypothermic exposure, while this process was effectively depressed by AFPIII [25]. This mechanism presumably led to the 80% survival rate of the cell after 72 h-preservation at 4°C, which used EC-solution containing 10 mg/ml of AFPIII [5].

The confocal photomicroscope images of HepG2 and RIN-5F cells (Figure 4) clearly showed that both AFPI and III are capable of binding to the surface of RIN-5F cells. The membrane surface was covered with AFP more entirely compared with BSA. Hence, taking all the obtained information together, we suggest that AFPI and III possess the same level of binding affinity to the surface of RIN-5F cells, covering the whole surface effectively to inhibit its swelling. This mechanism results in delaying of their rupture, which was detected as the improvement in survival rate.

A difference in the cell-preservation capacity between AFPI–III became evident in the present study; the ability of AFPII is poorer than that of AFPI and III (Figure 3). It should be noted that a size of the non-polar accessible surface area is approximately 2,400 Å^2^ for both AFPI and III, while it is 4,200 Å^2^ for AFPII [26] when evaluated for each protein’s coordinates (Protein data bank, http://www.rcsb.org/pdb/), 1WFB, 2ZIB, and 1HG7, respectively. Although it is unclear what prescribes the capacity of AFPI–III, the hydrophobicity specified with the non-polar accessible surface area might be one of the factors to differentiate AFPII from the others, since it will facilitate a proper binding of an AFP to the lipid bilayer. To clarify whether the ice-binding surface of AFPs shares the membrane-binding surface should be another interesting issue.

Insulin-dependent diabetes mellitus is a sickness that affects millions of people who have difficulty controlling their blood-sugar levels owing to deficiencies in the insulinoma cells [16]. Transplantation of insulinoma cells to such diabetic patients has been tried from the 1990s. Currently, the cells collected from a donor are all stored in a blood bag prior to being infused into a portal vein in the patient’s liver. When the insulinoma cells are bound to the portal vein, they work as a sensor to monitor the blood-sugar level and secrete insulin [27]. A key step is the quality storage of the insulinoma cells collected from a donor, to which AFP is expected to make a contribution. It is thought that AFPs do not present chemical toxicity threats at high concentrations (40 mg/ml) [28], and do not affect the cell osmotically due to their high molecular weight, and they are soluble in buffer solutions. With the helps of mass-preparation technique, AFP may enable 5-day quality storage of the insulinoma cells collected from a donor without freezing. This will lead to an improvement of a success rate of diabetes mellitus treatment.
